# New reverse sum Revan indices for physicochemical and pharmacokinetic properties of anti-filovirus drugs

**DOI:** 10.3389/fchem.2024.1486933

**Published:** 2024-12-19

**Authors:** W. Tamilarasi, B. J. Balamurugan

**Affiliations:** Department of Mathematics, School of Advanced Sciences, Vellore Institute of Technology, Chennai, Tamil Nadu, India

**Keywords:** anti-filovirus drugs, Ebola virus, Marburg virus, reverse sum Revan index, QSPR, multilinear regression, artificial neural network

## Abstract

Ebola and Marburg viruses, biosafety level 4 pathogens, cause severe hemorrhaging and organ failure with high mortality. Although some FDA-approved vaccines or therapeutics like Ervebo for Zaire Ebola virus exist, still there is a lack of effective therapeutics that cover all filoviruses, including both Ebola and Marburg viruses. Therefore, some anti-filovirus drugs such as Pinocembrin, Favipiravir, Remdesivir and others are used to manage infections. In theoretical chemistry, a chemical molecule is converted into an isomorphic molecular graph, G ( V,E ) by considering atom set V as vertices and bond set E as edges. A topological index is a molecular descriptor derived from the molecular graph of a chemical compound that characterizes its topology. The relationship between a compound’s chemical structure and its properties is investigated through the quantitative structure-property relationship (QSPR). This article introduces new reverse sum Revan degree based indices to explore the physicochemical and pharmacokinetic properties of anti-filovirus drugs via multilinear regression. The findings reveal a strong correlation between these proposed indices and the properties of anti-filovirus drugs when compared to reverse and Revan degree-based indices. Thus, reverse sum Revan indices offer valuable insights for analyzing the drugs properties used to treat Ebola and Marburg virus infections. Moreover, the multilinear regression (MLR) results through reverse sum Revan indices are compared with Artificial Neural Network (ANN) modelling technique and it provides the better prediction of the physicochemical and pharmacokinetic properties of anti-filovirus drugs.

## 1 Introduction

Marburg and Ebola virus infections are haemorrhagic fevers and highly contagious characterized by bleeding. They are caused by viruses called filoviruses. Ebola virus (EBOV) and Marburg virus (MARV) are lethal zoonotic virus which has been classified by the WHO as a pathogen in risk group 4 (WHO 2022) (Islam et al., 2023). The fatality rate for Marburg and Ebola viruses in Africa is typically 30%–60%, and can reach 90% where medical care is poor. The Marburg virus was first identified in 1967 in Germany. Since then, it has spread to other countries, including Guinea, Ghana, and Tanzania, creating public health concerns and attracting media attention to the disease (Marzi and Feldmann, 2023). As of 24 March 2023, Tanzania has reported five deaths among eight confirmed cases of Marburg virus disease. Common symptoms of both EBOV and MARV include high fever, headache, malaise, muscle aches, diarrhoea, abdominal discomfort, and cramping (Ahmed et al., 2023). Several vaccines to protect against EBOV and MARV are in the midst of clinical trials and antiviral treatments for Marburg virus are currently under development. The FDA-approved vaccine ‘Ervebo’ only protects against the Zaire Ebola virus and does not provide protection against other strains or filoviruses like the Marburg virus. Additionally, while monoclonal antibodies have been developed for treating Ebola, including some that target multiple strains, there is still no comprehensive vaccine or therapeutic option that effectively addresses all known filoviruses. However, some antifilovirus, such as Pinocembrin, Favipiravir, Tilorone, Quinacrine, Pyronaridine, Remdesivir, Galidesvir, Chloroquine, Clomiphene, Toremifene, Imatinib, Nilotinib, Amiloride, Paliperidone, Obeldesivir, Estradiol benzoate, Artemisinin, Ribavirin, Esomeprazole, Omeprazole, Tamoxifen, and 4-Aminomethyl benzamide show therapeutic efficacy against Marburg and Ebola virus.

Pinocembrin, also known as 5, 7-dihydroxy flavanone, is the flavonoid group of molecules available in natural sources and has numerous biological benefits such as antioxidant, antibacterial, antiviral, and anti-inflammatory functionalities (Hanieh et al., 2017; Shen et al., 2019). The pinocembrin derivatives demonstrated notable structural and pharmacological characteristics, making them potential candidates for antiviral treatments against Monkeypox and Marburg viruses (Akash et al., 2023). The repurposed drugs Tilorone, Quinacrine, and Pyronaridine, which inhibit the Marburg virus, also exhibit *in vitro* activity against SARS-CoV-2 and suggest potential mechanisms of action (Puhl et al., 2021). Remdesivir, an antiviral medicine for a variety of viruses, such as the Marburgvirus, the Ebolavirus, and more recently, the SARS-CoV-2 virus (Porter et al., 2020). The study in Zhu et al. (2018) suggests that Favipiravir emerges as a promising therapeutic candidate for MARV infection, particularly due to its potential for rapid and safe oral administration, making it valuable in outbreak scenarios, even post-exposure. As observed with Marburgvirus, galidesivir demonstrated strong *in vitro* antiviral activity against various ebolavirus species (Julander et al., 2021). Chloroquine could be utilized for treating filoviral infections as well as other viral infections that require an acidic pH for their infectivity (Akpovwa, 2016). The qRT-PCR results verified that clomiphene and toremifene were effective against both the native Ebola virus species and MARV (Johansen et al., 2013). Imatinib, Nilotinib, and Amiloride are among the FDA-approved drugs that may be used to treat Ebola virus infection (Yuan, 2015). Estradiol benzoate and paliperidone show potential as inhibitors of the VP35 protein of the Marburg virus, as identified through a cheminformatics approach (Srivastava et al., 2024). The anti-inflammatory property of artemisinin exhibit a potential antidisease therapeutic for Ebola patients in addition to its antimalarial activity (Haque et al., 2015). In cell culture, ribavirin was effective at reducing production of infectious EBOV (Alfson et al., 2015). Esomeprazole and omeprazole were also shown to inhibit viral entry in *in vitro* studies, providing protection against EBOV infection (Long et al., 2015). The inhibitors of 4-(aminomethyl)benzamide are also effective against Marburg virus (Gaisina et al., 2020).

Chemical graph theory is a branch of mathematical chemistry that studies graphs to represent and analyze chemical structures. A molecular structure is transformed into a molecular graph G ( V,E ) by considering atoms set V as vertices and bonds set E as edges. Topological indices are mathematical descriptors of molecular graphs to quantify the structural properties of molecules. The indices provide valuable information about molecular characteristics, such as connectivity, branching, symmetry, and other structural features. In addition to that they are widely used in various areas of chemistry, including drug design, chemical property prediction, and molecular modeling. These indices offer a method for numerical representation of molecular structure, which can subsequently be employed in quantitative structure-property relationship (QSPR) analysis.

Topological indices play an important role in drug development as they provide quantitative representations of molecular structures, which are valuable for predicting activity, evaluating drug-likeness, and estimating pharmacokinetic (ADMET) properties. Topological indices are employed to estimate various chemical-physical properties of molecules, such as boiling point, melting point, solubility, molecular weight, and lipophilicity. By correlating the structural features captured by the indices with experimental data, it is possible to develop models to explore these properties for new molecules. Absorption, distribution, metabolism, excretion, and toxicity (Admet) are crucial factors to consider in drug discovery. Topological indices can be utilized to explore various ADMET properties, such as Intestinal absorption, Skin permeability, BBB permeability, Water solubility, and metabolic stability. Caco-2 permeability is an important indicator of intestinal absorption and oral bioavailability, crucial for orally administered drugs. Higher permeability implies better absorption, enhancing systemic circulation and therapeutic effectiveness. For anti-filovirus drugs, compounds with high Caco-2 permeability could improve oral dosing and patient compliance. While blood-brain barrier (BBB) permeability is generally less critical for Ebola or Marburg viruses, it remains relevant for antiviral drugs in cases involving potential CNS complications. By estimating these properties early in the drug discovery process, researchers can prioritize molecules with favorable ADMET profiles and reduce the likelihood of failures in later stages of development. Overall, topological indices are crucial in chemical graph theory as they provide quantitative measures of molecular structures, which can be utilized for understanding, predicting, and designing chemical compounds.

Currently, researchers have introduced a wide range of indices which fall into the categories viz., degree-based, distance-based, eccentricity-based and temperature-based topological indices (Deutsch and Klavˇzar, 2014; Hu et al., 2021; Gao et al., 2018; Kansal et al., 2023). Out of all different types of topological indices, the degree based topological indices are frequently used indices in the QSPR/QSAR analysis to characterize the physicochemical and pharmokinetic properties of molecules. Recently in several articles (Mondal et al., 2022; Shanmukha et al., 2020; Kirmani et al., 2021; Zhong et al., 2021; Havare, 2021; Parveen et al., 2022; Tamilarasi and Balamurugan, 2022a; Tamilarasi and Balamurugan, 2022b; Tamilarasi and Balamurugan, 2023; Nasir et al., 2022; Zhang et al., 2022; Adnan et al., 2022; Kalaimathi and Balamurugan, 2023; Thilsath Parveen and Balamurugan, 2023; Balasubramaniyan and Chidambaram, 2023; Liu et al., 2021; Paul et al., 2023) QSPR analysis for various disease like COVID-19, cancer, malaria, monkeypox, rheumatoid arthritis, tuberculosis, asthma, Zika virus and fungal diseases are investigated using degree-based topological indices. This article introduces a new concept called the isomorphic molecular graph and a new index, namely, reverse sum Revan index are computed for anti-filovirus drugs. QSPR analysis are also performed using a multilinear regression equation to examine the physicochemical and pharmacokinetic properties of these drugs. Further, a comparison study were made to demonstrate how the reverse sum Revan indices differ significantly and improve upon the reverse and Revan degree-based indices. Additionally, we also compared multilinear regression with Artificial neural network model and exhibited that the multilinear regression model provide best prediction of physicochemical and pharmacokinetic properties of anti-filovirus drugs through reverse sum Revan indices when compared to ANN model.

**FIGURE 1:**
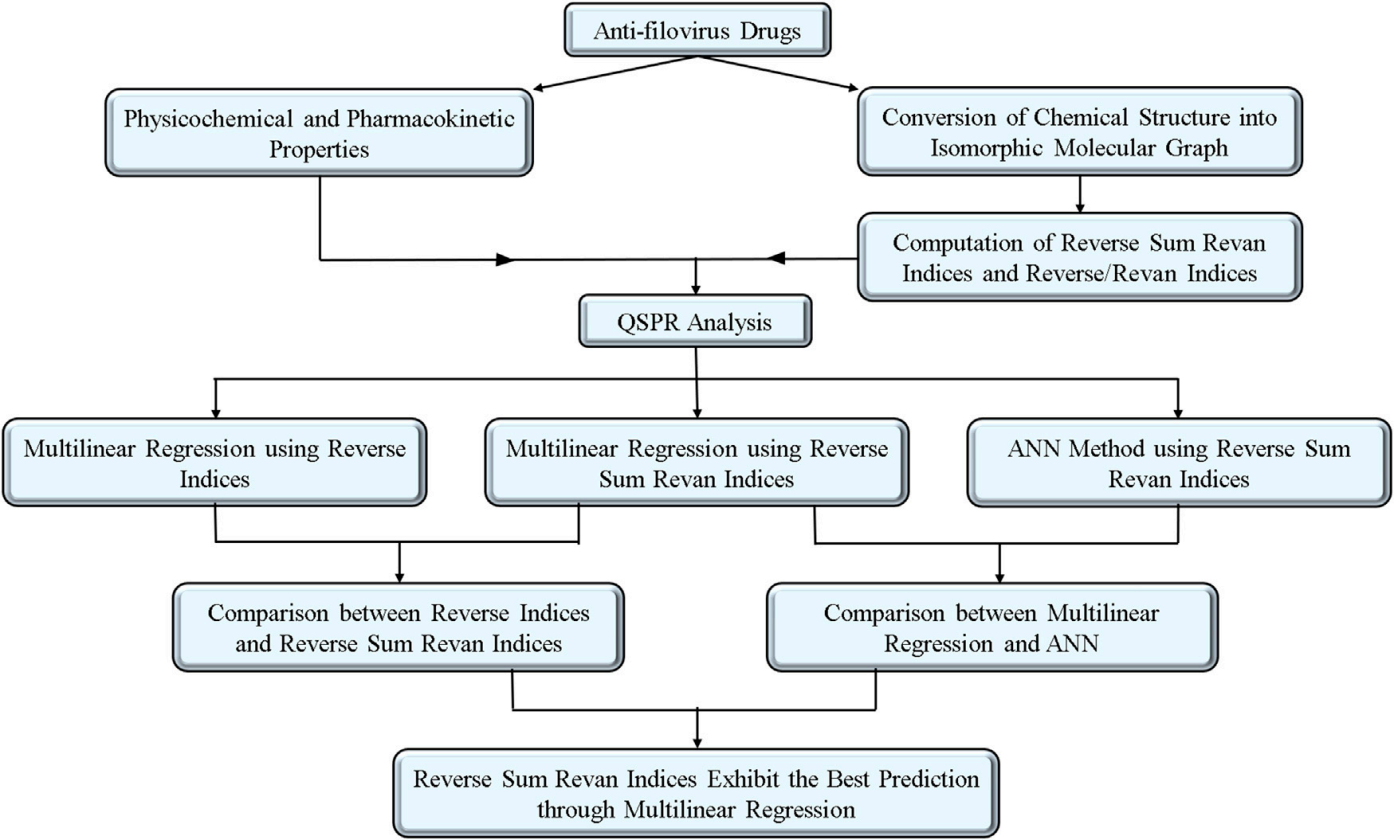
Workflow of the article.

The choice of drug compounds reflects their accessibility, use in current experimental therapies, and demonstrated antiviral potential. Many of these drugs have shown promise in earlier studies for inhibiting viral replication or exhibit pharmacokinetic properties favorable for therapeutic application. By analyzing this set, we aimed to investigate the structure-property relationships of drugs that are actively explored or potentially viable for filovirus treatment. To further enhance the impact of the drugs, Table 1 is included to summarize key pharmacokinetic and physicochemical properties of selected potential anti-filovirus drugs, supported by references to relevant studies for additional context. The methodology for computation of indices, QSPR analysis with comparative result between existing and proposed indices and comparison of MLR and ANN modelling technique are visualized in Figure 1.

**TABLE 1:** Key pharmacokinetic and physicochemical properties of anti-filovirus drugs.

Anti-filovirus drugs	Pharmacokinetic properties	Physicochemical properties	Reference
Pinocembrin	Absorbed quickly, distributed widely, easily pass through the BBB, anti-oxidative, anti-inflammatory	Low molecular weight, Good Lipophilicity	Hanieh et al. (2017), Shen et al. (2019a), Shen et al. (2019b)
Favipiravir	Plasma half-life, rapid and safe oral administration	Lipophilicity, Polarizability	Zhu et al. (2018), Mentré et al. (2015)
Tilorone	Excellent solubility, high Caco2 permeability, high maximum tolerated dose	Metabolic stability	Puhl et al. (2021), Ekins et al. (2018)
Quinacrine	Permeable to the blood -brain barrier	Membrane fluidity	Sarangi et al. (2022)
Pyronaridine	Rapid oral absorption, low total body clearance and exhibit *in vitro* activity against SARS-CoV-2	Large volume of distribution, molecular weight, higher aqueous solubility	Chu and Dorlo (2023)
Remdesivir	High intracellular concentration and antiviral medicine	Low molecular weight, improved physicochemical stability	Porter et al. (2020), Humeniuk et al. (2021)
Chloroquine	Anti-inflammatory effect, binding affinity, rapid absorption, extreme toxic	Electrostatic potential, high volume of distribution	Akpovwa (2016), Askarian et al. (2022)
Nilotinib	Reduces inflammation, good permeability of BBB	Increase dopamine level	Yuan, 2015; Pagan et al. (2019)
Ribavirin	Low BBB value, negative skin permeability and reduces production of infectious EBOV	Lipophilicity and water solubility	Alfson et al. (2015), chauhan et al. (2019)
Esomeprazole	Rapid metabolism well tolerated dose and inhibit viral entry in *in vitro* studies	Soluble in organic solvent	Long et al. (2015), Kim et al. (2023)

## 2 Isomorphic molecular graphs and reverse sum revan degree-based indices

This section introduces a new concept called the isomorphic chemical graphs, which is used to explore the structural properties of anti-filovirus drugs. Additionally, a new topological descriptor, the reverse sum Revan topological index, has been introduced, based on a novel vertex degree for the isomorphic molecular graph $G\left(V,E\right)$ .

### 2.1 Motivation

Wiener (Wiener, 1947) introduced two topological descriptors called Wiener index and Polarity index. Using these indices, Wiener predicted the boiling point of alkanes, where the chemical structure of alkanes contains only the single bond. Computing the Wiener index for the molecular graph of a chemical compound formed by considering double and triple bonds as a single edge does not affect the index value, as the Wiener index is a distance-based topological descriptor. Moreover, Wiener, excluded hydrogen atoms to simplify molecular graphs by focusing on atom connectivity. His index captures key structural features by measuring distances between atom pairs, avoiding the added complexity of hydrogen atoms. Later, many studies have been carried out by researchers on various topological indices for the prediction of properties of the chemical compounds by considering hydrogen depleted molecular graph and the double bond and triple bond as a single edge. In Gutman and Polansky (2012) Ivan Gutman and Oskar E. Polansky introduced a concept known as complete molecular graph, including all hydrogen atoms in the molecular framework but still considering double and triple bonds as a single edge.

Many researchers construct molecular graphs by converting double and triple bonds into a single edge instead of multiple edges. However, this approach is not acceptable from a chemist’s perspective and goes against graph theory principles. In our research, we compute the degree-based topological indices of molecular graphs and these indices are mainly computed by the degree of atoms. Hence, in this article, the chemical structures are converted into isomorphic chemical graphs by incorporating, double bonds as two parallel edges, triple bonds as three parallel edges and hydrogen and other atoms are preserved in their adjacency. These isomorphic chemical graphs will stimulate accurate representation of chemical structure of a molecule. This approach preserves the unique structural characteristics of the molecule, allowing for accurate comparison and analysis.

The choice of using the reverse sum Revan index instead of reverse degree-based index and Revan index is that this index has ability to provide a more comprehensive, sensitive, and refined measure of isomorphic molecular graph of chemical molecule. By combining the strengths of both the reverse degree approach and the Revan index methodology, the reverse sum Revan index offers enhanced correlation with molecular properties, better applicability to complex networks, and a robust, interpretable framework for advanced research applications. This makes it a valuable tool for researchers seeking detailed insights into the structure and properties of complex systems.

The reverse sum Revan degree based indices were developed to address specific limitations of traditional indices, namely, lower predictive accuracy and reduced interpretability for anti-filovirus drugs. Existing indices often lack sensitivity to subtle structural variations that impact drug efficacy, limiting their ability to model complex structure-property relationships. The new indices enhance predictive power by capturing finer details in molecular topology, making them more effective and interpretable for drug development against Ebola and Marburg viruses.

Definition 1: Let M be a molecular structure of a chemical molecule. The isomorphic molecular graph of M is a graph $G\left(V,E\right)$ formed by considering atom set V as vertices and bond set E as edges. The double, triple bonds in M are considered as two and three parallel edges respectively and vertex set V includes hydrogen atoms in isomorphic molecular graph $G\left(V,E\right)$ .

**FIGURE 2:**
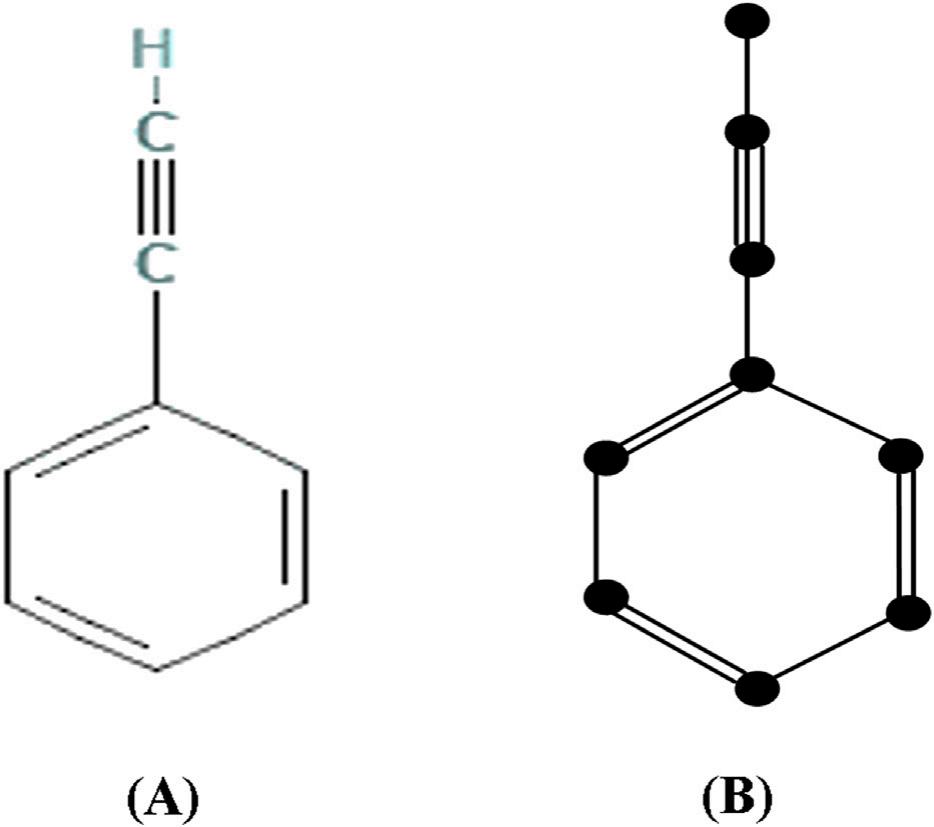
**(A)** Chemical structure and **(B)** Isomorphic chemical graph of Phenylacetylene.

The following Figure 2 depicts the chemical structure and isomorphic molecular graph of Phenylacetylene.

V.R.Kulli in (Kulli, 2018) defined the concept of reverse vertex degree $r_{u}$ of a vertex $u\in V\left(G\right)$ as

$$r_{u}=∆\left(G\right)-d_{G}\left(u\right)+1$$

In Kulli (2017) V.R.Kulli, introduced another vertex degree, namely, Revan vetex degree $R_{u}$ of a vertex $u\in V\left(G\right)$ as

$$R_{u}=∆\left(G\right)+\delta \left(G\right)-d_{G}\left(u\right)$$

where $∆\left(G\right)$ and $\delta \left(G\right)$ denote the maximum and minimum degree of a graph G and $d_{G}\left(u\right)$ denote the degree of a vertex $u\in V\left(G\right)$ . Based on these two we inroduced a new vertex degree namely, reverse sum Revan vertex degree which is defined as follows:

Definition 2: Let G be a isomorphic molecular graph with atom set V ( $\left.G\right)$ and bond set E ( G ). The reverse sum Revan vertex degree ${rR}_{u}$ of an atom u in G is defined as

$${rR}_{u}=2∆\left(G\right)+\delta \left(G\right)-2d_{G}\left(u\right)+1$$

where $∆\left(G\right)$ and $\delta \left(G\right)$ denote the maximum and minimum degree of the isomorrphic molecular graph G and $d_{G}\left(u\right)$ denote the degree of an atom $u\in V\left(G\right)$ .

Example 1: The reverse sum Revan vertex degrees of all the atoms for the isomorphic molecular graph of acetaminophen with maximum degree eight and minimum degree 2 with their molecular structure are provided in Supplementary Material. The number of atoms and bonds in acetaminophen are $\left|V\left(A\right)\right|=13$ and $\left|E\left(A\right)\right|=17$ .

The reverse sum Revan vertex degree are defined for the following indices: the first and second Zagreb index (Gutman et al., 1975; Austel et al., 1983), first and second Hyper Zagreb index (Došlić et al., 2011a; Došlić et al., 2011b), Forgotten index (Furtula and Gutman, 2015), Atom Bond Connectivity index (Estrada et al., 1998), Geometric Arithmetic index (Vukičević and Furtula, 2009), redefined first, second and third Zagreb index (Ranjini et al., 2013). Accordingly, the reverse sum Revan indices with their mathematical formulae are represented in Table 2.

**TABLE 2:** Reverse sum Revan indices and their mathematical formulas.

S. No	Reverse sum revan indices	Notation	Mathematical formula
I.	Reverse sum Revan first Zagreb index	$rRM_{1}\left(G\right)$	$\sum_{uv\in E\left(G\right)}\left[rR\left(u\right)+rR\left(v\right)\right]$
II.	Reverse sum Revan second Zagreb index	$rRM_{2}\left(G\right)$	$\sum_{uv\in E\left(G\right)}\left[rR\left(u\right)\times rR\left(v\right)\right]$
III.	Reverse sum Revan first Hyper Zagreb index	$rR{HM}_{1}\left(G\right)$	$\sum_{uv\in E\left(G\right)}{\left[rR\left(u\right)+rR\left(v\right)\right]}^{2}$
IV.	Reverse sum Revan second Hyper Zagreb index	$rR{HM}_{2}\left(G\right)$	$\sum_{uv\in E\left(G\right)}{\left[rR\left(u\right)\times rR\left(v\right)\right]}^{2}$
V.	Reverse sum Revan Forgotten index	$rRF\left(G\right)$	$\sum_{uv\in E\left(G\right)}\left[rR{\left(u\right)}^{2}+rR{\left(v\right)}^{2}\right]$
VI.	Reverse sum Revan Atom Bond Connectivity index	$rRABC\left(G\right)$	$\sum_{uv\in E\left(G\right)}\frac{\left[rR\left(u\right)+rR\left(v\right)-2\right]}{\left[rR\left(u\right)\times rR\left(v\right)\right]}$
VII.	Reverse sum Revan Geometric Arithmetic index	$rRGA\left(G\right)$	$\sum_{uv\in E\left(G\right)}\frac{2\sqrt{rR\left(u\right)\times rR\left(v\right)}}{\left[rR\left(u\right)+rR\left(v\right)\right]}$
VIII.	Reverse sum Revan redefined first Zagreb index	$rReZG_{1}\left(G\right)$	$\sum_{uv\in E\left(G\right)}\frac{\left[rR\left(u\right)+rR\left(v\right)\right]}{\left[rR\left(u\right)\times rR\left(v\right)\right]}$
IX.	Reverse sum Revan redefined second Zagreb index	$rReZG_{2}\left(G\right)$	$\sum_{uv\in E\left(G\right)}\frac{\left[rR\left(u\right)\times rR\left(v\right)\right]}{\left[rR\left(u\right)+rR\left(v\right)\right]}$
X.	Reverse sum Revan redefined third Zagreb index	$rReZG_{3}\left(G\right)$	$\sum_{uv\in E\left(G\right)}\left[rR\left(u\right)+rR\left(v\right)\right]\left[rR\left(u\right)\times rR\left(v\right)\right]$

## 3 Computation of reverse sum revan indices of anti-filovirus drugs

The molecular structures of Pinocembrin, Favipiravir, Tilorone, Quinacrine, Pyronaridine, Remdesivir, Galidesvir, Chloroquine, Clomiphene, Toremifene, Imatinib, Nilotinib, Amiloride, Paliperidone, Obeldesivir, Estradiol benzoate, Artemisinin, Ribavirin, Esomeprazole, Omeprazole, Tamoxifen and 4-Aminomethyl benzamide are provided in the Supplementary Material. Using the reverse sum Revan degree-based edge partition, the indices provided in Table 2 are computed for the isomorphic chemical graphs of these drugs.

Theorem 1 The reverse sum Revan indices for the isomorphic chemical graph P of Pinocembrin are given by

(i). $rRM_{1}\left(P\right)=224$ (vi). $rRABC\left(P\right)=13.25$

(ii). $rRM_{2}\left(P\right)=424$ (vii). $rRGA\left(P\right)=28.6$

(iii). $rR{HM}_{1}\left(P\right)=1848$ (viii). $rReZG_{1}\left(P\right)=19.5833$

(iv). $rR{HM}_{2}\left(P\right)=9376$ (ix). $rReZG_{2}\left(P\right)=51.0571$

(v). $rRF\left(P\right)=1000$ (x). $\mathrm{rReZ}G_{3}\left(P\right)=3904$

**FIGURE 3:**
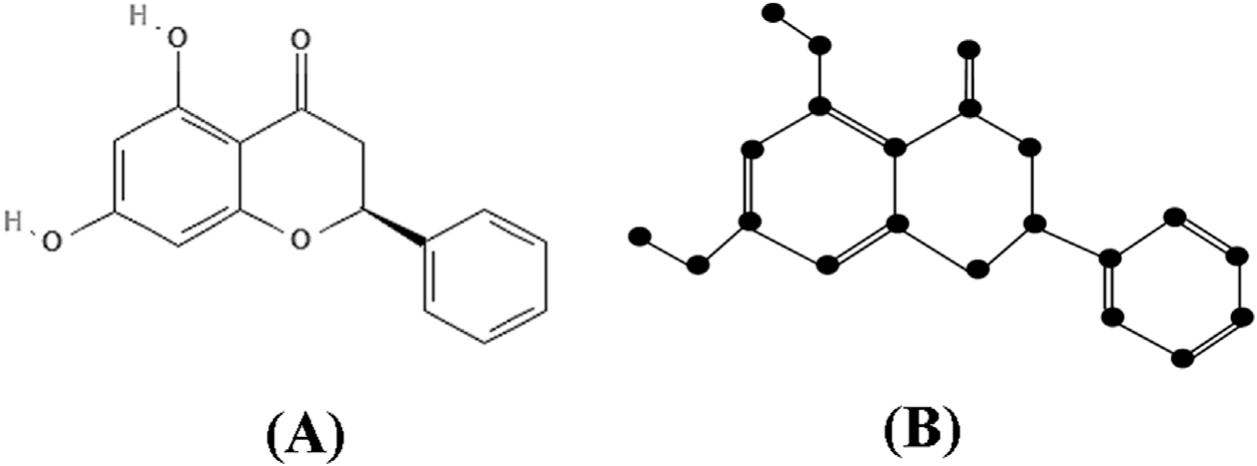
**(A)** Chemical structure and **(B)** Isomorphic chemical graph of Pinocembrin.

Proof: The molecular structure and isomorphic chemical graph of Pinocembrin are depicted in Figures 3A, B respectively. The number of atoms and bonds of the isomorphic chemical graph P are $\left|V\left(P\right)\right|=21$ and $\left|E\left(P\right)\right|=30$ . The edge partition of the isomorphic molecular graph P of Pinocembrin based on reverse sum Revan degree are given in Table 3.

By analyzing the graph structure and using the edge partition in Table 3 the indices values are computed as follows:

(i). $rRM_{1}\left(P\right)\begin{array}{c} =\sum_{uv\in E\left(P\right)}\left[rR\left(u\right)+rR\left(v\right)\right]\\ =2\left(8+6\right)+3\left(6+4\right)+6\left(6+2\right)+6\left(4+4\right)+9\left(4+2\right)+4\left(2+2\right)\\ =224 \end{array}$

(ii). $\begin{array}{cc} rRM_{2}\left(P\right) & \begin{array}{c} =\sum_{uv\in E\left(P\right)}\left[rR\left(u\right)\times rR\left(v\right)\right]\\ =2\left(8\times 6\right)+3\left(6\times 4\right)+6\left(6\times 2\right)+6\left(4\times 4\right)+9\left(4\times 2\right)+4\left(2\times 2\right)\\ =424 \end{array} \end{array}$

(iii). $rR{HM}_{1}\left(P\right)\begin{array}{c} =\sum_{uv\in E\left(P\right)}{\left[rR\left(u\right)+rR\left(v\right)\right]}^{2}\\ =2{\left(14\right)}^{2}+3{\left(10\right)}^{2}+6{\left(8\right)}^{2}+6{\left(8\right)}^{2}+9{\left(6\right)}^{2}+4{\left(4\right)}^{2}\\ =1848 \end{array}$

(iv). $rR{\text{HM}}_{2}\left(P\right)\begin{array}{c} =\sum_{uv\in E\left(P\right)}{\left[rR\left(u\right)\times rR\left(v\right)\right]}^{2}\\ =2{\left(48\right)}^{2}+3{\left(24\right)}^{2}+6{\left(12\right)}^{2}+6{\left(16\right)}^{2}+9{\left(8\right)}^{2}+4{\left(4\right)}^{2}\\ =9676 \end{array}$

(v). $rRF\left(P\right)\begin{array}{c} =\sum_{uv\in E\left(P\right)}\left[rR{\left(u\right)}^{2}+rR{\left(v\right)}^{2}\right]\\ =2\left(64+36\right)+3\left(36+16\right)+6\left(36+4\right)+6\left(16+16\right)+9\left(16+4\right)+4\left(4+4\right)\\ =1000 \end{array}$

(vi). $rRABC\left(P\right)\begin{array}{c} =\sum_{uv\in E\left(P\right)}\frac{\left[rR\left(u\right)+rR\left(v\right)-2\right]}{\left[rR\left(u\right)\times rR\left(v\right)\right]}\\ =2\left(\frac{12}{48}\right)+3\left(\frac{8}{24}\right)+6\left(\frac{6}{12}\right)+6\left(\frac{6}{16}\right)+9\left(\frac{4}{8}\right)+4\left(\frac{2}{4}\right)\\ =13.25 \end{array}$

(vii). $rRGA\left(P\right)\begin{array}{c} =\sum_{uv\in E\left(P\right)}\frac{2\sqrt{rR\left(u\right)\times rR\left(v\right)}}{\left[rR\left(u\right)+rR\left(v\right)\right]}\\ =2\left(\frac{2\sqrt{48}}{14}\right)+3\left(\frac{2\sqrt{24}}{10}\right)+6\left(\frac{2\sqrt{12}}{8}\right)+6\left(\frac{2\sqrt{16}}{8}\right)+9\left(\frac{2\sqrt{8}}{6}\right)+4\left(\frac{2\sqrt{4}}{4}\right)\\ =28.6 \end{array}$

(viii). $rReZG_{1}\left(P\right)\begin{array}{c} =\sum_{uv\in E\left(P\right)}\frac{\left[rR\left(u\right)+rR\left(v\right)\right]}{\left[rR\left(u\right)\times rR\left(v\right)\right]}\\ =2\left(\frac{8+6}{8\times 6}\right)+3\left(\frac{6+4}{6\times 4}\right)+6\left(\frac{6+2}{6\times 2}\right)+6\left(\frac{4+4}{4\times 4}\right)+9\left(\frac{4+2}{4\times 2}\right)+4\left(\frac{2+2}{2\times 2}\right)\\ =19.5833 \end{array}$

(ix). $rReZG_{2}\left(P\right)\begin{array}{c} =\sum_{uv\in E\left(P\right)}\frac{\left[rR\left(u\right)\times rR\left(v\right)\right]}{\left[rR\left(u\right)+rR\left(v\right)\right]}\\ =2\left(\frac{8\times 6}{8+6}\right)+3\left(\frac{6\times 4}{6+4}\right)+6\left(\frac{6\times 2}{6+2}\right)+6\left(\frac{4\times 4}{4+4}\right)+9\left(\frac{4\times 2}{4+2}\right)+4\left(\frac{2\times 2}{2+2}\right)\\ =51.0571 \end{array}$

(x). $rReZG_{3}\left(P\right)\begin{array}{c} =\sum_{uv\in E\left(P\right)}\left[rR\left(u\right)+rR\left(v\right)\right]\left[rR\left(u\right)\times rR\left(v\right)\right]\\ =2\left(14\right)\left(48\right)+3\left(10\right)\left(24\right)+6\left(8\right)\left(12\right)+6\left(8\right)\left(16\right)+9\left(6\right)\left(8\right)+4\left(4\right)\left(4\right)\\ =3904 \end{array}$

Similarly, the reverse sum Revan indices are computed for other drugs considered in this research in same manner as in Theorem 1. The computed indices values are provided in Tables 4, 5.

**TABLE 3:** Reverse sum Revan degree-based edge partition of Pinocembrin.

$\left({rR}_{P}\left(u\right),{rR}_{P}\left(v\right)\right)/$ $uv\in E\left(P\right)$	(8,6)	(6,4)	(6,2)	(4,4)	(4,2)	(2,2)
No. of edges	2	3	6	6	9	4

**TABLE 4:** Reverse sum Revan degree based indices( $rRM_{1},rRM_{2},rR{HM}_{1},rR{HM}_{2},rRF$ ) of anti-filovirus drugs.

Drugs	$rRM_{1}$	$rRM_{2}$	$rR{HM}_{1}$	$rR{HM}_{2}$	rRF
Pinocembrin	224	424	1848	9,376	1,000
Favipiravir	136	240	1,136	4,640	656
Tilorone	316	700	2,944	20,208	1,544
Quinacrine	296	620	2,648	16,528	1,300
Pyronaridine	408	820	3,512	20,208	1872
Remdesivir	736	2,220	9,556	99,312	4,856
Galidesvir	272	600	2,560	16,736	1,360
Chloroquine	244	504	2,216	12,832	1,208
Clomiphene	312	624	2,616	14,016	1,368
Toremifene	320	640	2,680	14,048	1,400
Imatinib	402	740	3,212	13,488	1732
Nilotinib	410	668	3,036	9,456	1700
Amiloride	216	416	1912	9,632	1,080
Paliperidone	322	660	2,868	15,952	1,548
Obeldesivir	312	640	2,768	15,616	1,488
Estradiol benzoate	320	592	2,688	12,256	1,504
Artemisinin	228	428	2080	9,360	1,224
Ribavirin	236	552	2,320	15,744	1,216
Esomeprazole	244	420	1928	8,784	1,048
Omeprazole	244	420	1928	8,784	1,048
Tamoxifen	300	588	2,472	12,496	1,296
4-Aminomethyl benzamide	152	296	1,328	6,336	736

**TABLE 5:** Reverse sum Revan degree based indices ( $rRABC,rRGA,rReZG_{1},rReZG_{2},rReZG_{3}$ ) of anti-filovirus drugs.

Drugs	rRABC	rRGA	$rReZG_{1}$	$rReZG_{2}$	$rReZG_{3}$
Pinocembrin	13.25	28.6	19.5833	51.0571	3,904
Favipiravir	8.3125	16.6921	12.166	28.933	2,192
Tilorone	16.111	37.7297	24.165	74.4472	7,344
Quinacrine	16.138	36.6382	24.248	68.984	6,272
Pyronaridine	23.4785	51.8639	35.4993	94.5231	7,968
Remdesivir	18.662	59.2128	22.7976	174.81	29,520
Galidesvir	13.395	38.964	19.83	63.745	6,272
Chloroquine	12.737	28.884	18.403	55.347	5,008
Clomiphene	17.175	40.227	25.284	74.157	5,760
Toremifene	17.538	40.893	25.537	79.158	5,872
Imatinib	23.66	51.711	34.19	91.455	6,336
Nilotinib	27.25	55.892	40.208	91.333	5,200
Amiloride	12.5	26.328	18.705	47.26	4,064
Paliperidone	17.048	38.09	24.691	73.488	6,193
Obeldesivir	16.482	37.331	23.733	71.789	6,272
Estradiol benzoate	18.743	39.561	27.704	70.628	5,424
Artemisinin	12.291	24.418	17.246	46.63	4,192
Ribavirin	9.812	24.908	13.368	55.945	5,840
Esomeprazole	16.4375	32.916	25.208	53.383	3,792
Omeprazole	16.4375	32.916	25.208	53.383	3,792
Tamoxifen	16.888	39.388	24.956	71.158	5,312
4-Aminomethyl benzamide	8.205	17.947	11.666	33.899	2,800

## 4 Computation of reverse and revan degree based topological indices for anti-filovirus drugs

In this section we compute the reverse and Revan degree based topological indices for the anti-filovirus drugs mentioned in section 3, in order to compare newly introduced reverse sum Revan indices with existing indices. All isomorphic molecular graphs of filovirus drugs considered in this article have minimum degree δ=1. Therefore the reverse vertex degree and Revan vertex degree are same for all the drugs. Hence it is enough to compute either reverse topological indices or Revan topological indices. Using the reverse degree-based edge partition and indices formulas in (Kulli, 2018) the reverse degree based indices are computed for the isomorphic molecular graphs of anti-filovirus drugs and the values are listed in Tables 7, 8.

Theorem 2 The reverse degree based indices for the isomorphic chemical graph P of Pinocembrin are given by

(i). $rM_{1}\left(P\right)=110$ (vi). $rABC\left(P\right)=13.833$

(ii). $rM_{2}\left(P\right)=102$ (vii). $\text{rGA}\left(P\right)=28.562$

(iii). $r{HM}_{1}\left(P\right)=446$ (viii). $reZG_{1}\left(P\right)=39.833$

(iv). $r{HM}_{2}\left(P\right)=554$ (ix). $reZG_{2}\left(P\right)=24.995$

(v). $rF\left(P\right)=250$ (x). $reZG_{3}\left(P\right)=464$

Proof: The molecular structure and isomorphic chemical graph P of Pinocembrin are shown in Figures 3A, B respectively. The number of atoms and bonds of P are $\left|V\left(P\right)\right|=21$ and $\left|E\left(P\right)\right|=30.$ The edge partition of the isomorphic molecular graph P of Pinocembrin based on reverse degree are given in Table 6.

By analyzing the graph structure and using the edge partition in Table 6 the indices values are computed as follows:

(i). ${rM}_{1}\left(P\right)\begin{array}{c} =\sum_{uv\in E\left(P\right)}\left[r\left(u\right)+r\left(v\right)\right]\\ =4\left(1+1\right)+10\left(1+2\right)+6\left(1+3\right)+6\left(2+2\right)+2\left(2+3\right)+2\left(3+4\right)\\ =110 \end{array}$

(ii). $rM_{2}\left(P\right)\begin{array}{c} =\sum_{\text{uv}\in E\left(P\right)}\left[r\left(u\right)\times r\left(v\right)\right]\\ =4\left(1\times 1\right)+10\left(1\times 2\right)+6\left(1\times 3\right)+6\left(2\times 2\right)+2\left(2\times 3\right)+2\left(3\times 4\right)\\ =102 \end{array}$

(iii). $r{HM}_{1}\left(P\right)\begin{array}{c} =\sum_{uv\in E\left(P\right)}{\left[r\left(u\right)+r\left(v\right)\right]}^{2}\\ =4{\left(2\right)}^{2}+10{\left(3\right)}^{2}+6{\left(4\right)}^{2}+6{\left(4\right)}^{2}+2{\left(5\right)}^{2}+2{\left(7\right)}^{2}\\ =446 \end{array}$

(iv). $r{HM}_{2}\left(P\right)\begin{array}{c} =\sum_{uv\in E\left(P\right)}{\left[r\left(u\right)\times r\left(v\right)\right]}^{2}\\ =4{\left(1\right)}^{2}+10{\left(2\right)}^{2}+6{\left(3\right)}^{2}+6{\left(4\right)}^{2}+9{\left(6\right)}^{2}+4{\left(12\right)}^{2}\\ =554 \end{array}$

(v). $rF\left(P\right)\begin{array}{c} =\sum_{uv\in E\left(P\right)}\left[r{\left(u\right)}^{2}+r{\left(v\right)}^{2}\right]\\ =4\left(1+1\right)+10\left(1+4\right)+6\left(1+9\right)+6\left(4+4\right)+2\left(4+9\right)+2\left(9+16\right)\\ =250 \end{array}$

(vi). $rABC\left(P\right)\begin{array}{c} =\sum_{uv\in E\left(P\right)}\frac{\left[r\left(u\right)+r\left(v\right)-2\right]}{\left[r\left(u\right)\times r\left(v\right)\right]}\\ =10\left(\frac{1}{2}\right)+6\left(\frac{2}{3}\right)+6\left(\frac{2}{4}\right)+2\left(\frac{3}{6}\right)+2\left(\frac{5}{12}\right)\\ =13.833 \end{array}$

(vii). $rGA\left(P\right)\begin{array}{c} =\sum_{uv\in E\left(P\right)}\frac{2\sqrt{r\left(u\right)\times r\left(v\right)}}{\left[r\left(u\right)+r\left(v\right)\right]}\\ =4\left(\frac{2\sqrt{1}}{2}\right)+10\left(\frac{2\sqrt{2}}{3}\right)+6\left(\frac{2\sqrt{3}}{4}\right)+6\left(\frac{2\sqrt{4}}{4}\right)+2\left(\frac{2\sqrt{6}}{5}\right)+2\left(\frac{2\sqrt{12}}{7}\right)\\ =28.562 \end{array}$

(viii). $reZG_{1}\left(P\right)\begin{array}{c} =\sum_{\text{uv}\in E\left(P\right)}\frac{\left[r\left(u\right)+r\left(v\right)\right]}{\left[r\left(u\right)\times r\left(v\right)\right]}\\ =4\left(\frac{1+1}{1\times 1}\right)+10\left(\frac{1+2}{1\times 2}\right)+6\left(\frac{1+3}{1\times 3}\right)+6\left(\frac{2+2}{2\times 2}\right)+2\left(\frac{2+3}{2\times 3}\right)+2\left(\frac{3+4}{3\times 4}\right)\\ =39.833 \end{array}$

(ix). $reZG_{2}\left(P\right)\begin{array}{c} =\sum_{uv\in E\left(P\right)}\frac{\left[r\left(u\right)\times r\left(v\right)\right]}{\left[r\left(u\right)+r\left(v\right)\right]}\\ =4\left(\frac{1\times 1}{1+1}\right)+10\left(\frac{1\times 2}{1+2}\right)+6\left(\frac{1\times 3}{1+3}\right)+6\left(\frac{2\times 2}{2+2}\right)+2\left(\frac{2\times 3}{2+3}\right)+2\left(\frac{3\times 4}{3+4}\right)\\ =24.995 \end{array}$

(x). $reZG_{3}\left(P\right)\begin{array}{c} =\sum_{uv\in E\left(P\right)}\left[r\left(u\right)+r\left(v\right)\right]\left[r\left(u\right)\times r\left(v\right)\right]\\ =4\left(2\right)\left(1\right)+10\left(3\right)\left(2\right)+6\left(4\right)\left(3\right)+6\left(4\right)\left(4\right)+2\left(5\right)\left(6\right)+2\left(7\right)\left(12\right)\\ =464 \end{array}$

Similarly, the reverse degree based indices are computed for other drugs considered in this research in same manner as in Theorem 2. The computed indices values are provided in Tables 7, 8.

**TABLE 6:** Reverse degree-based edge partition of Pinocembrin.

$\left(r_{P}\left(u\right),r_{P}\left(v\right)\right)/$ $uv\in E\left(P\right)$	(1,1)	(1,2)	(1,3)	(2,2)	(2,3)	(3,4)
No. of edges	4	10	6	6	2	2

**TABLE 7:** Reverse degree based indices( $rM_{1},rM_{2},r{HM}_{1},r{HM}_{2},rF$ ) of anti-filovirus drugs.

Drugs	$rM_{1}$	$rM_{2}$	$r{HM}_{1}$	$r{HM}_{2}$	rF
Pinocembrin	110	102	446	554	250
Favipiravir	68	60	284	290	164
Tilorone	150	169	704	1,245	366
Quinacrine	145	153	653	1,029	341
Pyronaridine	196	201	858	1,263	456
Remdesivir	344	526	2,204	6,008	1,152
Galidesvir	136	150	640	1,046	340
Chloroquine	122	126	554	802	302
Clomiphene	156	156	654	876	342
Toremifene	160	160	670	878	350
Imatinib	201	185	803	843	433
Nilotinib	205	167	759	591	425
Amiloride	108	104	478	602	270
Paliperidone	161	165	717	997	387
Obeldesivir	156	160	692	976	372
Estradiol benzoate	160	148	672	766	376
Artemisinin	114	107	520	585	306
Ribavirin	118	138	580	984	304
Esomeprazole	122	105	482	549	272
Omeprazole	122	105	482	549	272
Tamoxifen	150	147	618	781	324
4-Aminomethyl benzamide	76	74	332	396	184

**TABLE 8:** Reverse degree based indices( $rABC,rGA,reZG_{1},reZG_{2},reZG_{3}$ ) of anti-filovirus drugs.

Drugs	rABC	rGA	$reZG_{1}$	$reZG_{2}$	$reZG_{3}$
Pinocembrin	13.83	28.56	39.83	24.99	464
Favipiravir	8.92	16.69	24.33	14.47	274
Tilorone	14.78	36.00	45.67	35.72	894
Quinacrine	15.56	35.70	46.99	33.81	778
Pyronaridine	21.17	49.09	66.00	45.59	913
Remdesivir	26.63	46.42	41.23	81.58	3,538
Galidesvir	13.94	31.96	39.67	31.88	784
Chloroquine	14.14	28.55	36.83	26.43	632
Clomiphene	18.14	39.90	38.58	37.08	720
Toremifene	18.58	40.90	51.58	38.08	734
Imatinib	25.97	51.71	68.42	46.22	792
Nilotinib	28.58	55.90	80.42	45.67	650
Amiloride	12.58	26.33	37.42	23.63	514
Paliperidone	18.81	38.09	49.42	36.75	806
Obeldesivir	18.44	37.33	47.50	35.90	784
Estradiol benzoate	19.58	39.56	55.42	35.31	678
Artemisinin	14.67	24.42	34.50	22.82	524
Ribavirin	12.53	25.26	26.75	27.98	730
Esomeprazole	15.33	32.92	50.42	26.70	474
Omeprazole	15.33	32.92	50.42	26.70	474
Tamoxifen	17.64	38.90	49.92	35.58	664
4-Aminomethyl benzamide	9.50	17.95	23.33	16.95	350

## 5 QSPR analysis for properties of anti-filovirus drugs through multilinear regression and reverse sum revan indices

In this section QSPR analysis is carried out for the physicochemical and pharmacokinetic properties of anti-filovirus through multilinear regression and reverse sum Revan degree based indices.

### 5.1 Methodology

The quantitative structure-property relationship (QSPR) analysis is conducted between the computed reverse sum Revan topological indices and the physicochemical and pharmacokinetic properties of anti-filovirus drugs. The physicochemical and pharmacokinetic properties of Pinocembrin, Favipiravir, Tilorone, Quinacrine, Pyronaridine, Remdesivir, Galidesvir, Chloroquine, Clomiphene, Toremifene, Imatinib, Nilotinib, Amiloride, Paliperidone, Obeldesivir, Estradiol benzoate, Artemisinin, Ribavirin, Esomeprazole, Omeprazole, Tamoxifen and 4-Aminomethyl benzamide are provided in Tables 9, 10. The physicochemical properties considered for this analysis are Molar Refractivity (MR), Polarizability (P), Molar Volume (MV), Molecular weight (MW), Heavy Atom Count (HAC), Complexity (C), Monoisotopic Mass (MM) and lipophilicity (LogP). The physicochemical properties of the drugs are sourced from the chemical structure database ChemSpider (https://www.chemspider.com). The pharmacokinetic properties considered for study are Caco2 permeability, BBB permeability, CNS permeability, Maximum tolerated dose and Minnow Toxicity. The pharmacokinetic properties of the drugs are obtained using the platform pkCSM/ADMET.

**TABLE 9:** Physicochemical properties of anti-filovirus drugs.

Drugs	MR	P	MV	MW	HAC	C	MM	LogP
Pinocembrin	68.4	27.1	184.8	256.25	19	337	256.07	3.93
Favipiravir	33.2	13.2	97.2	157.10	11	282	157.03	0.78
Tilorone	121.1	48	372.4	410.5	30	477	410.26	5.25
Quinacrine	122	48.3	346	436.4	29	461	399.21	4.85
Pyronaridine	150.8	59.8	381.8	518	37	707	517.22	4.80
Remdesivir	149.5	59.3	409	602.6	42	1,010	602.23	2.10
Galidesvir	68.3	27.1	162.6	265.27	19	334	265.12	−1.73
Chloroquine	97.4	38.6	287.9	319.9	22	309	319.18	4.81
Clomiphene	123.7	49.1	367.6	405.9	29	481	405.19	6.56
Toremifene	123.7	49.1	367.6	405.9	29	483	405.19	6.21
Imatinib	147.1	58.3	393	493.6	37	706	493.26	4.59
Nilotinib	141.8	56.2	388.7	529.5	39	817	529.18	6.35
Amiloride	49.9	19.8	108.6	229.6	15	279	229.05	−1.29
Paliperidone	112.6	44.6	294.1	426.4	31	764	426.21	3.08
Obeldesivir	88	34.9	226.8	361.3	26	595	361.14	−0.64
Estradiol benzoate	109.3	43.3	317.6	376.4	28	582	376.20	5.12
Artemisinin	70.3	27.9	226.5	282.3	20	452	282.15	2.39
Ribavirin	51.1	20.3	117.1	244.2	17	304	244.08	−3.01
Esomeprazole	94	37.3	251.9	345.4	24	453	345.11	2.89
Omeprazole	94	37.3	251.9	345.4	24	453	345.11	2.89
Tamoxifen	118.9	47.1	356.2	371.5	28	463	371.22	5.99
4-Aminomethyl benzamide	43.6	17.3	128.1	150.1	11	139	150.08	0.24

**TABLE 10:** Pharmacokinetic properties of anti-filovirus drugs.

Drugs	Caco2 permeability	BBB permeability	CNS permeability	Max.tolerated dose	Minnow toxicity
Pinocembrin	1.15	0.42	−2.05	0.27	1.68
Favipiravir	0.62	−0.13	−3.08	1.29	3.41
Tilorone	1.04	−0.11	−2.25	0.27	1.32
Quinacrine	0.98	0.03	−1.48	0.73	0.98
Pyronaridine	0.97	−1.26	−2.30	0.44	−1.34
Remdesivir	0.64	−2.06	−4.68	0.15	0.29
Galidesvir	−0.75	−1.57	−5.16	0.49	4.14
Chloroquine	1.62	0.35	−2.19	−0.17	0.75
Clomiphene	0.94	1.21	−1.37	0.46	0.37
Toremifene	0.97	1.27	−1.34	0.36	1.09
Imatinib	1.09	−0.38	−2.51	0.32	2.09
Nilotinib	1.39	−0.68	−2.05	0.20	1.30
Amiloride	−0.39	−1.00	−3.66	0.94	3.70
Paliperidone	1.29	−0.11	−2.35	−0.69	0.76
Obeldesivir	0.66	−1.15	−3.75	0.14	2.64
Estradiol benzoate	1.16	−0.10	−1.31	−0.05	0.42
Artemisinin	1.30	0.24	−2.91	0.07	1.41
Ribavirin	0.42	−0.92	−3.76	1.01	4.63
Esomeprazole	1.29	−0.29	−2.62	0.50	−0.36
Omeprazole	1.29	−0.29	−2.62	0.50	−0.36
Tamoxifen	1.07	1.33	−1.47	0.31	0.60
4-Aminomethyl benzamide	0.76	0.38	−2.89	0.62	2.48

The physicochemical and pharmacokinetic properties are often influenced by multiple factors, such as molecular weight, solubility, polarity, and many other molecular descriptors. A multiple linear regression model allows us to include several independent variables, capturing the combined effect of these factors on the dependent variable. In contrast, linear, quadratic, and cubic regressions typically involve a single independent variable, which may not fully capture the complexity of the relationships in this context. Notably, the multiple linear regression analysis yielded significant results with a lower standard error and high coefficient value compared to the linear, quadratic, and cubic regression models for these indices. A general multiple linear regression equaion is

$$P=\beta_{0}+\beta_{1}T_{1}+\beta_{2}T_{2}+\ldots +\beta_{p}T_{p}$$

where

• P is the dependent variable representing the physicochemical and pharmacokinetic properties.
• $T_{1}$ , $T_{2},\ldots ,T_{p}$ are the independent variables representing the reverse sum Revan indices.
• $\beta_{0}$ is the intercept (constant term).
• $\beta_{1}$ $,\ldots ,\beta_{p}$ are the coefficients (slopes) corresponding to each independent variable.

is employed in QSPR to analyze the relationship between one dependent variable (response variable) and several independent variables (predictor variables).

The main statistical parameters required in QSPR analysis are the Root Mean Square Error (RMSE) and the $R^{2}$ (coefficient of determination) value. The $R^{2}$ value measures the proportion of variance in the dependent variable that is predictable from the independent variables and RMSE represents the square root of the average of squared differences between the observed and predicted values. The value of $R^{2}$ lies in the range of 0–1. A multilinear regression with high $R^{2}$ value and low RMSE is generally considered as good fit. The SPSS software is employed to evaluate the $R^{2}$ and RMSE values through Euqations 1, 2 in order to get error free results.

$$R^{2}=1-\frac{\sum_{i=1}^{n}{\left(y_{i}-\hat{y_{i}}\right)}^{2}}{\sum_{i=1}^{n}{\left(y_{i}-\bar{y}\right)}^{2}}$$ (1)

$$RMSE=\sqrt{\frac{1}{n}\sum_{i=1}^{n}{\left(y_{i}-\hat{y_{i}}\right)}^{2}}$$ (2)

where $y_{i}$ is actual observed value for the ith data point, $\hat{y_{i}}$ is predicted value from the model for the ith data point, $\bar{y}$ is mean of the observed values and n is number of observations.

### 5.2 Multilinear regressions for physicochemical properties of filovirus drugs through reverse sum revan indices

The best fit multilinear regression Equations 3–10 for the physicochemical properties of filovirus drugs, which are obtained through QSPR analysis are presented in this section. The statistical parameters corresponding to the regression lines are summarized in Table 11.

$$MR=-6.969+0.566\left(rRM_{2}\right)+0.001\left(rR{HM}_{2}\right)-0.066\left(rRF\right)-9.591\left(rRABC\right)-3.085\left(rRGA\right)+10.556\left(rReZG_{1}\right)+0.445\left(rReZG_{2}\right)-0.030\left(rReZG_{3}\right)$$ (3)

$$P=-2.727+0.221\left(rRM_{2}\right)+0.001\left(rR{HM}_{2}\right)-0.026\left(rRF\right)-3.840\left(rRABC\right)-1.223\left(rRGA\right)+4.194\left(rReZG_{1}\right)+0.194\left(rReZG_{2}\right)-0.012\left(rReZG_{3}\right)$$ (4)

$$MV=-7.529+0.561\left(rRM_{2}\right)+0.002\left(rR{HM}_{2}\right)-0.122\left(rRF\right)-38.983\left(rRABC\right)-12.620\left(rRGA\right)+33.991\left(rReZG_{1}\right)+9.691\left(rReZG_{2}\right)-0.049\left(rReZG_{3}\right)$$ (5)

$$MW=4.526+2.310\left(rRM_{2}\right)+0.000\left(rR{HM}_{2}\right)-0.242\left(rRF\right)+4.902\left(rRABC\right)-9.205\left(rRGA\right)+17.800\left(rReZG_{1}\right)-5.930\left(rReZG_{2}\right)-0.078\left(rReZG_{3}\right)$$ (6)

$$HAC=-0.872+0.178\left(rRM_{2}\right)+0.001\left(rR{HM}_{2}\right)-0.012\left(rRF\right)+0.421\left(rRABC\right)-0.555\left(rRGA\right)+0.994\left(rReZG_{1}\right)-0.385\left(rReZG_{2}\right)-0.010\left(rReZG_{3}\right)$$ (7)

$$C=-87.758+6.276\left(rRM_{2}\right)+0.074\left(rR{HM}_{2}\right)-0.390\left(rRF\right)+45.607\left(rRABC\right)-9.771\left(rRGA\right)-1.524\left(rReZG_{1}\right)-22.276\left(rReZG_{2}\right)-0.626\left(rReZG_{3}\right)$$ (8)

$$MM=1.923+1.996\left(rRM_{2}\right)+0.009\left(rR{HM}_{2}\right)-0.092\left(rRF\right)-11.257\left(rRABC\right)-8.579\left(rRGA\right)+23.283\left(rReZG_{1}\right)-2.755\left(rReZG_{2}\right)-0.121\left(rReZG_{3}\right)$$ (9)

$$LogP=-0.790-0.033\left(rRM_{2}\right)+0.002\left(rR{HM}_{2}\right)+0.005\left(rRF\right)-1.533\left(rRABC\right)-0.501\left(rRGA\right)+1.095\left(rReZG_{1}\right)+0.743\left(rReZG_{2}\right)-0.007\left(rReZG_{3}\right)$$ (10)

**TABLE 11:** Highly correlated statistical parameters for physicochemical properties through MLR and reverse sum Revan index.

Physicochemical properties	R	$R^{2}$	RMSE
MR	0.961	0.923	12.69428
P	0.981	0.963	3.64659
MV	0.948	0.898	44.14312
MW	0.984	0.968	26.76138
HAC	0.988	0.976	1.67835
C	0.972	0.945	61.39844
MM	0.987	0.974	23.88981
LogP	0.766	0.587	2.32452

From Table 11, it is observed that except for lipophilicity (LogP), all other properties are well predicted using the multilinear regression equations since they exhibit high $R^{2}$ value with lower the value of RMSE.

### 5.3 Multilinear regressions for pharmacokinetic properties of filovirus drugs through reverse sum revan indices

The best fit multilinear regression Equations 11–15 for the pharmacokinetic properties of filovirus drugs, which are obtained through QSPR analysis are presented in this section. The statistical parameters corresponding to the regression lines are summarized in Table 12.

$$Caco2=0.813+0.017\left(rRM_{2}\right)+0.000\left(rR{HM}_{2}\right)-0.001\left(rRF\right)+0.600\left(rRABC\right)-0.181\left(rRGA\right)-0.163\left(rReZG_{1}\right)+0.003\left(rReZG_{2}\right)-0.002\left(rReZG_{3}\right)$$ (11)

$$BBB=1.177-0.018\left(rRM_{2}\right)+0.001\left(rR{HM}_{2}\right)+0.001\left(rRF\right)-0.250\left(rRABC\right)-0.166\left(rRGA\right)-0.019\left(rReZG_{1}\right)+0.341\left(rReZG_{2}\right)-0.003\left(rReZG_{3}\right)$$ (12)

$$CNS=-2.93-0.003\left(rRM_{2}\right)+0.000\left(rR{HM}_{2}\right)-4.364E-5\left(rRF\right)-0.670\left(rRABC\right)-0.345\left(rRGA\right)+0.538\left(rReZG_{1}\right)+0.295\left(rReZG_{2}\right)-0.002\left(rReZG_{3}\right)$$ (13)

$$MTD=0.973-0.026\left(rRM_{2}\right)+0.000\left(rR{HM}_{2}\right)-0.005\left(rRF\right)+0.572\left(rRABC\right)+0.003\left(rRGA\right)-0.281\left(rReZG_{1}\right)+0.043\left(rReZG_{2}\right)+0.004\left(rReZG_{3}\right)$$ (14)

$$MT=3.323-0.008\left(rRM_{2}\right)-0.001\left(rR{HM}_{2}\right)-0.005\left(rRF\right)+2.062\left(rRABC\right)+0.352\left(rRGA\right)-1.576\left(rReZG_{1}\right)-0.209\left(rReZG_{2}\right)-0.005\left(rReZG_{3}\right)$$ (15)

**TABLE 12:** Highly correlated statistical parameters for pharmacokinetic properties through MLR and reverse sum Revan index.

Pharmacokinetic properties	R	$R^{2}$	RMSE
Caco2 permeability	0.804	0.647	0.43488
BBB permeability	0.781	0.610	0.70498
CNS permeability	0.836	0.699	0.73604
Max.tolerated dose	0.768	0.591	0.33918
Minnow toxicity	0.816	0.665	1.12539

From Table 12, it is observed that except maximum tolerated dose all other properties are well predicted using the multilinear regression equations since they exhibit high ${\;R}^{2}$ value with lower the value of RMSE. The weaker correlation for the maximum tolerated dose is probably due to the natural differences in toxicity data, which can be affected by factors like differences between species and how drugs behave in specific situations. One limitation of our analysis is that it depends on available physicochemical and pharmacokinetic data, which might not fully reflect how drugs interact, spread in the body, or are broken down in real-life conditions. Also, differences in how experiments are conducted and reported across studies could create inconsistencies in the data.

## 6 Multilinear regression analysis of filovirus drugs using reverse degree based topological indices

### 6.1 Multilinear regressions for physicochemical properties of filovirus drugs

The best fit multilinear regression Equations 16–23 for the physicochemical properties of filovirus drugs, which are obtained through QSPR analysis of reverse indices are presented in this section. The statistical parameters corresponding to the regression lines are summarized in Table 13.

$$MR=-10.740+12.861\left(rM_{1}\right)+1.332\left(rM_{2}\right)+0.076\left(r{HM}_{2}\right)-2.326\left(rF\right)-9.825\left(rABC\right)-0.751\left(reZG_{1}\right)-31.438\left(reZG_{2}\right)+0.032\left(reZG_{3}\right)$$ (16)

$$P=-4.172+5.095\left(rM_{1}\right)+0.523\left(rM_{2}\right)+0.031\left(r{HM}_{2}\right)-0.920\left(rF\right)-3.875\left(rABC\right)-0.302\left(reZG_{1}\right)+12.430\left(reZG_{2}\right)+0.012\left(reZG_{3}\right)$$ (17)

$$MV=-30.699+48.615\left(rM_{1}\right)+2.319\left(rM_{2}\right)+0.173\left(r{HM}_{2}\right)-8.705\left(rF\right)-35.428\left(rABC\right)-0.002\left(rGA\right)-4.687\left(reZG_{1}\right)-116.511\left(reZG_{2}\right)+0.599\left(reZG_{3}\right)$$ (18)

$$MW=-11.948+28.521\left(rM_{1}\right)+2.694\left(rM_{2}\right)+0.136\left(r{HM}_{2}\right)-4.919\left(rF\right)-21.674\left(rABC\right)-0.001\left(rGA\right)-0.207\left(reZG_{1}\right)-70.276\left(reZG_{2}\right)+0.158\left(reZG_{3}\right)$$ (19)

$$HAC=-2.184+1.684\left(rM_{1}\right)+0.233\left(rM_{2}\right)+0.006\left(r{HM}_{2}\right)-0.302\left(rF\right)-1.086\left(rABC\right)-3.043E-5\left(rGA\right)+0.009\left(reZG_{1}\right)-4.204\left(reZG_{2}\right)-0.007\left(reZG_{3}\right)$$ (20)

$$C=-144.655-4.683\left(rM_{1}\right)+0.869\left(rM_{2}\right)-0.010\left(r{HM}_{2}\right)+2.475\left(rF\right)+13.992\left(rABC\right)+0.001\left(rGA\right)+3.396\left(reZG_{1}\right)+8.737\left(reZG_{2}\right)-0.480\left(reZG_{3}\right)$$ (21)

$$MM=70.783+25.886\left(rM_{1}\right)+2.619\left(rM_{2}\right)+0.169\left(r{HM}_{2}\right)-4.337\left(rF\right)-18.777\left(rABC\right)-0.001\left(rGA\right)-0.440\left(reZG_{1}\right)-63.075\left(reZG_{2}\right)-0.006\left(reZG_{3}\right)$$ (22)

$$LogP=-2.523+1.863\left(rM_{1}\right)-0.054\left(rM_{2}\right)+0.017\left(r{HM}_{2}\right)-0.315\left(rF\right)-1.168\left(rABC\right)+3.127E-5\left(rGA\right)-0.269\left(reZG_{1}\right)-4.209\left(reZG_{2}\right)+0.011\left(reZG_{3}\right)$$ (23)

**TABLE 13:** Highly correlated statistical parameters for physicochemical properties through MLR and reverse index.

Physicochemical properties	R	$R^{2}$	RMSE
MR	0.981	0.963	3.64659
P	0.961	0.923	5.02696
MV	0.904	0.818	56.70508
MW	0.984	0.968	27.81872
HAC	0.985	0.971	1.95070
C	0.950	0.903	85.29742
MM	0.987	0.974	24.96415
LogP	0.844	0.713	2.01616

### 6.2 Multilinear regressions for pharmacokinetic properties of filovirus drugs

The best fit multilinear regression Equations 24–28 for the pharmacokinetic properties of filovirus drugs, which are obtained through QSPR analysis of reverse indices are presented in this section. The statistical parameters corresponding to the regression lines are summarized in Table 14.

$$Caco2=0.669+0.306\left(rM_{1}\right)+0.116\left(rM_{2}\right)+0.005\left(r{HM}_{2}\right)-0.075\left(rF\right)+0.224\left(rABC\right)+6.372E-6\left(rGA\right)-0.011\left(reZG_{1}\right)-1.197\left(reZG_{2}\right)-0.005\left(reZG_{3}\right)$$ (24)

$$BBB=0.545+0.405\left(rM_{1}\right)-0.020\left(rM_{2}\right)+0.002\left(r{HM}_{2}\right)-0.083\left(rF\right)-0.088\left(rABC\right)+1.178E-5\left(rGA\right)-0.112\left(reZG_{1}\right)-0.891\left(reZG_{2}\right)+0.009\left(reZG_{3}\right)$$ (25)

$$CNS=-3.452+0.543\left(rM_{1}\right)-0.007\left(rM_{2}\right)+0.005\left(r{HM}_{2}\right)-0.095\left(rF\right)-0.324\left(rABC\right)+1.162E-5\left(rGA\right)-0.093\left(reZG_{1}\right)-1.215\left(reZG_{2}\right)+0.002\left(reZG_{3}\right)$$ (26)

$$MTD=1.830-0.162\left(rM_{1}\right)-0.053\left(rM_{2}\right)+0.005\left(r{HM}_{2}\right)+0.026\left(rF\right)+0141\left(rABC\right)-2.193E-5\left(rGA\right)-0.001\left(reZG_{1}\right)+0.616\left(reZG_{2}\right)-0.006\left(reZG_{3}\right)$$ (27)

$$MT=3.347-0.981\left(rM_{1}\right)+0.036\left(rM_{2}\right)-0.021\left(r{HM}_{2}\right)+0.131\left(rF\right)+0.869\left(rABC\right)+3.388E-5\left(rGA\right)+0.121\left(reZG_{1}\right)+2.157\left(reZG_{2}\right)+0.024\left(reZG_{3}\right)$$ (28)

**TABLE 14:** Highly correlated statistical parameters for pharmacokinetic properties through MLR and reverse index.

Pharmacokinetic properties	R	$R^{2}$	RMSE
Caco2 permeability	0.787	0.620	0.43362
BBB permeability	0.767	0.589	0.75312
CNS permeability	0.758	0.575	0.90999
Max.tolerated dose	0.757	0.573	0.36044
Minnow toxicity	0.865	0.749	1.01492

## 7 Comparison analysis between new reverse sum revan index against existing reverse degree based index

**FIGURE 4:**
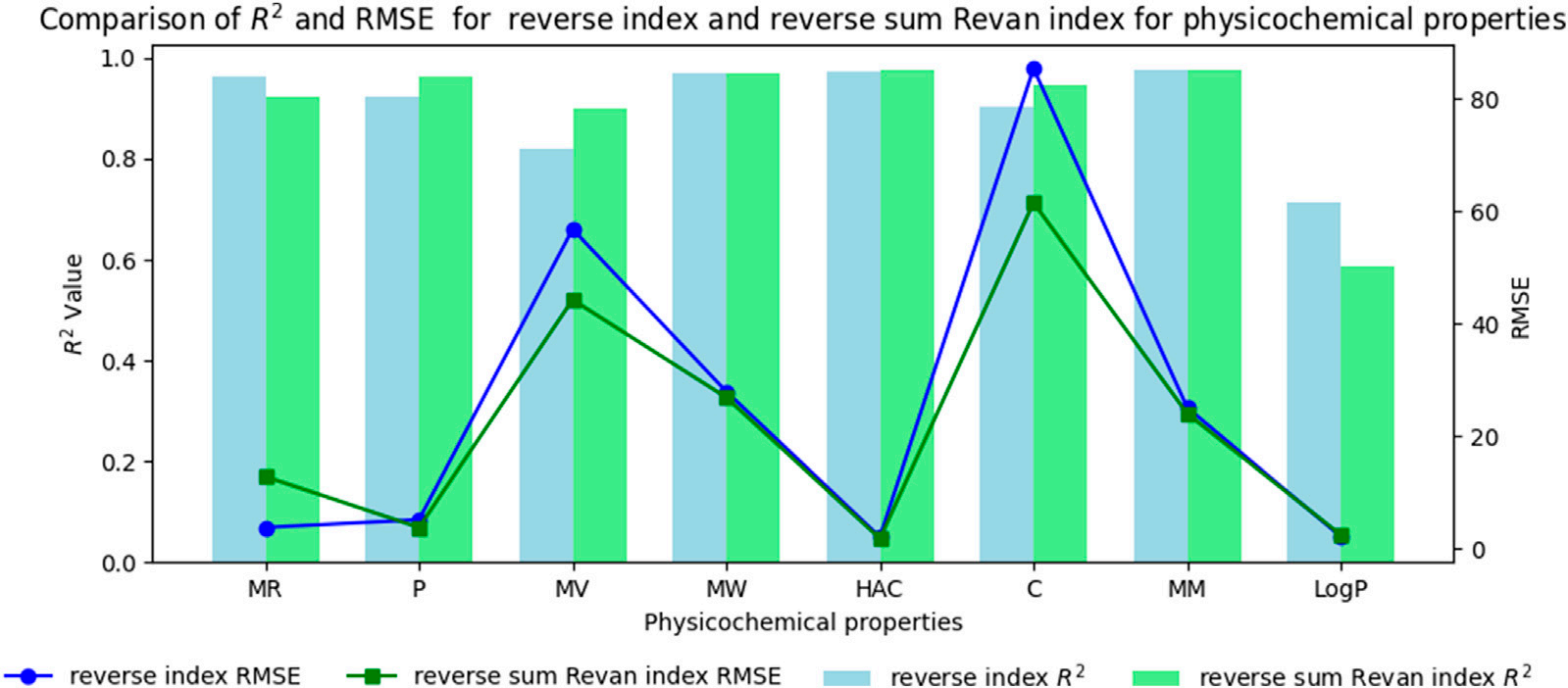
Comparison graph between existing reverse index and new reverse sum Revan index for physicochemical properties of filovirus drugs.

**FIGURE 5:**
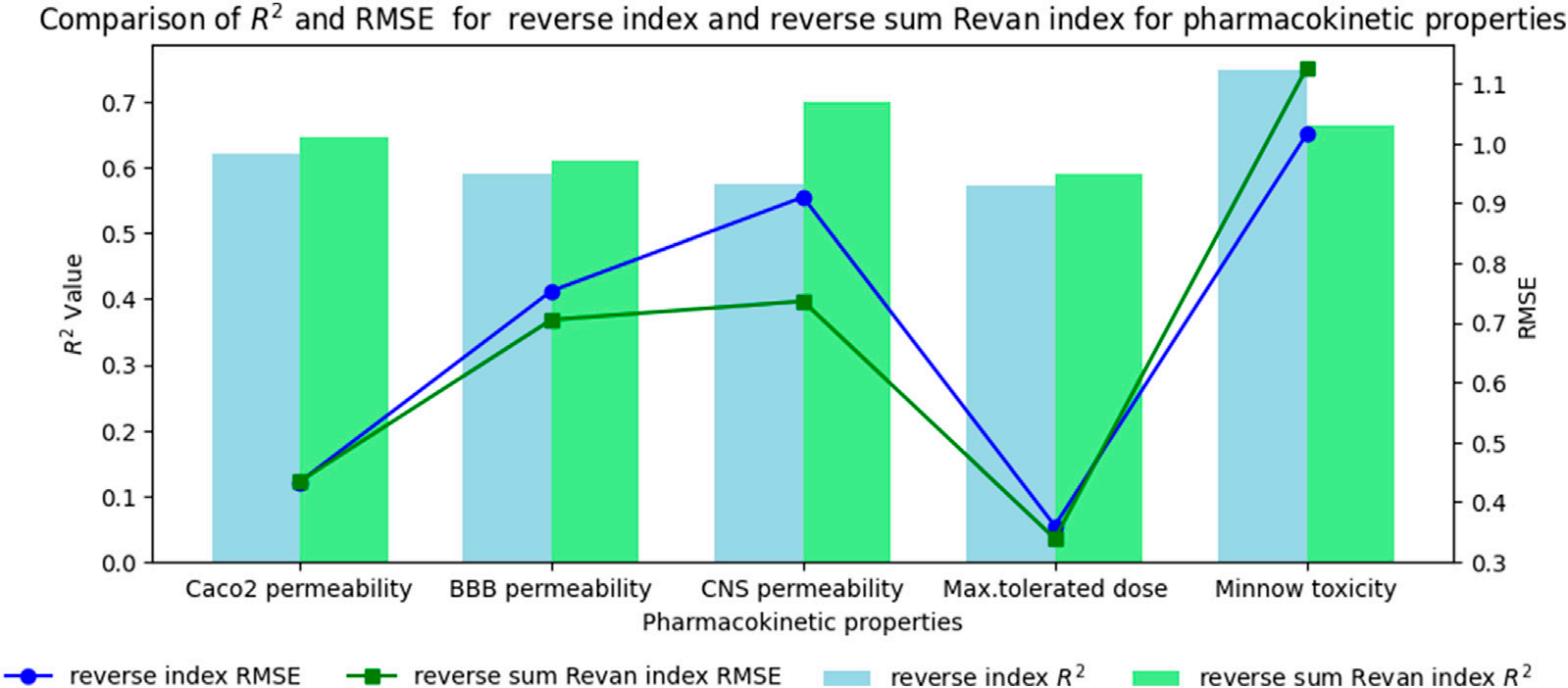
Comparison graph between existing reverse index and new reverse sum Revan index for pharmacokinetic properties of filovirus drugs.

In this section we compare the predictive capability of newly introduced reverse sum Revan index against the existing reverse degree based index. Tables 15, 16 shows the comparison of best predictive multilinear regressions for physicochemical and pharmacokinetic properties of filovirus drugs. The comparison graph between existing reverse index and new reverse sum Revan index are shown in Figures 4, 5.

**TABLE 15:** Comparison of best predictive fits for physicochemical properties between reverse sum Revan index and reverse index.

Physicochemical properties	MLR equations through reverse index	$R^{2}$	RMSE	MLR equations through reverse sum revan index	$R^{2}$	RMSE
MR	Equation 16	0.963	3.64659	Equation 3	0.923	12.69428
P	Equation 17	0.923	5.02696	Equation 4	0.963	3.64659
MV	Equation 18	0.818	56.70508	Equation 5	0.898	44.14312
MW	Equation 19	0.968	27.81872	Equation 6	0.968	26.76138
HAC	Equation 20	0.971	1.95070	Equation 7	0.976	1.67835
C	Equation 21	0.903	85.29742	Equation 8	0.945	61.39844
MM	Equation 22	0.974	24.96415	Equation 9	0.974	23.88981
LogP	Equation 23	0.713	2.01616	Equation 10	0.587	2.32452

**TABLE 16:** Comparison of best predictive fits for pharmacokinetic properties between reverse sum Revan index and reverse index.

Pharmacokinetic properties	MLR equations through reverse index	$R^{2}$	RMSE	MLR equations through reverse sum revan index	$R^{2}$	RMSE
Caco2 permeability	Equation 24	0.620	0.43362	Equation 11	0.647	0.43488
BBB permeability	Equation 25	0.589	0.75312	Equation 12	0.610	0.70498
CNS permeability	Equation 26	0.575	0.90999	Equation 13	0.699	0.73604
Max.tolerated dose	Equation 27	0.573	0.36044	Equation 14	0.591	0.33918
Minnow toxicity	Equation 28	0.749	1.01492	Equation 15	0.665	1.12539

### 7.1 Results and discussion

Based on the comparison in Tables 15, 16 between newly introduced reverse sum Revan index and existing reverse degree based index for physicochemical and pharmacokinetic properties the following results are obtained through QSPR analysis.

(i). Molar Refractivity (MR): The existing reverse index outperformed the new reverse sum Revan index with a higher $R^{2}$ of 0.963 and a lower RMSE of 3.65. The new index achieved a lower $R^{2}$ of 0.923 and higher RMSE of 12.69, indicating less predictive accuracy for this property.
(ii). Polarizability (P): The reverse sum Revan index showed superior performance with an $R^{2}$ of 0.963 and an RMSE of 3.65 compared to the existing index ( $R^{2}$ = 0.923, RMSE = 5.03), demonstrating its effectiveness for polarizability.
(iii). Molar Volume (MV): The reverse sum Revan index had a better $R^{2}$ = 0.898 and a significantly lower RMSE = 44.14 than the existing index ( $R^{2}$ = 0.818, RMSE = 56.71), indicating improved predictive capability.
(iv). Molecular Weight (MW) and Monoisotopic Mass (MM): Both indices showed comparable performance, with lower RMSEs, suggesting better precision.
(v). Heavy Atom Count (HAC) and Complexity (C): The new reverse sum Revan index outperformed the existing index in both $R^{2}$ and RMSE, showing a stronger predictive fit for these properties.
(vi). LogP: The existing reverse index was more effective, with an $R^{2}$ of 0.713 and a lower RMSE = 2.02 compared to the new index, which had an $R^{2}$ of 0.587 and RMSE of 2.32, indicating limitations of the reverse sum Revan index for this property.
(vii). Caco2 Permeability: The reverse sum Revan index showed a slightly higher $R^{2}$ = 0.647 compared to the existing reverse index ( $R^{2}$ = 0.620), indicating a marginally better predictive performance, though the RMSE was comparable.
(viii). BBB Permeability: The new reverse sum Revan index demonstrated improved performance with an $R^{2}$ of 0.610 and a lower RMSE = 0.705 compared to the existing index ( $R^{2}$ = 0.589, RMSE = 0.753).
(ix). CNS Permeability: The new index outperformed the existing one, achieving an $R^{2}$ of 0.699 and a reduced RMSE = 0.736, compared to the existing index ( $R^{2}$ = 0.575, RMSE = 0.910).
(x). Maximum Tolerated Dose: The reverse sum Revan index had a higher $R^{2}$ = 0.591 and a lower RMSE = 0.339 compared to the existing index ( $R^{2}$ = 0.573, RMSE = 0.360), suggesting a better fit.
(xi). Minnow Toxicity: The existing index performed better, achieving an ${\;R}^{2}$ of 0.749 and a lower RMSE of 1.015, while the new index had a lower $R^{2}$ (=0.665) and higher RMSE (=1.125), indicating reduced predictive power for toxicity.

It is observed that the multilinear regressions based on new reverse sum Revan indices provide the best predictive fits for most of the physicochemical and pharmacokinetic properties except Molar Refractivity, LogP and Minnow toxicity. Therefore, our new reverse sum Revan indices differ significantly and improve upon the existing reverse and Revan degree based indices.

## 8 Comparison of multilinear regression (MLR) with artificial neural network (ANN) modelling technique for physicochemical and pharmacokinetic properties of filovirus drugs through reverse sum revan indices

### 8.1 Artificial neural network (ANN)

**FIGURE 6:**
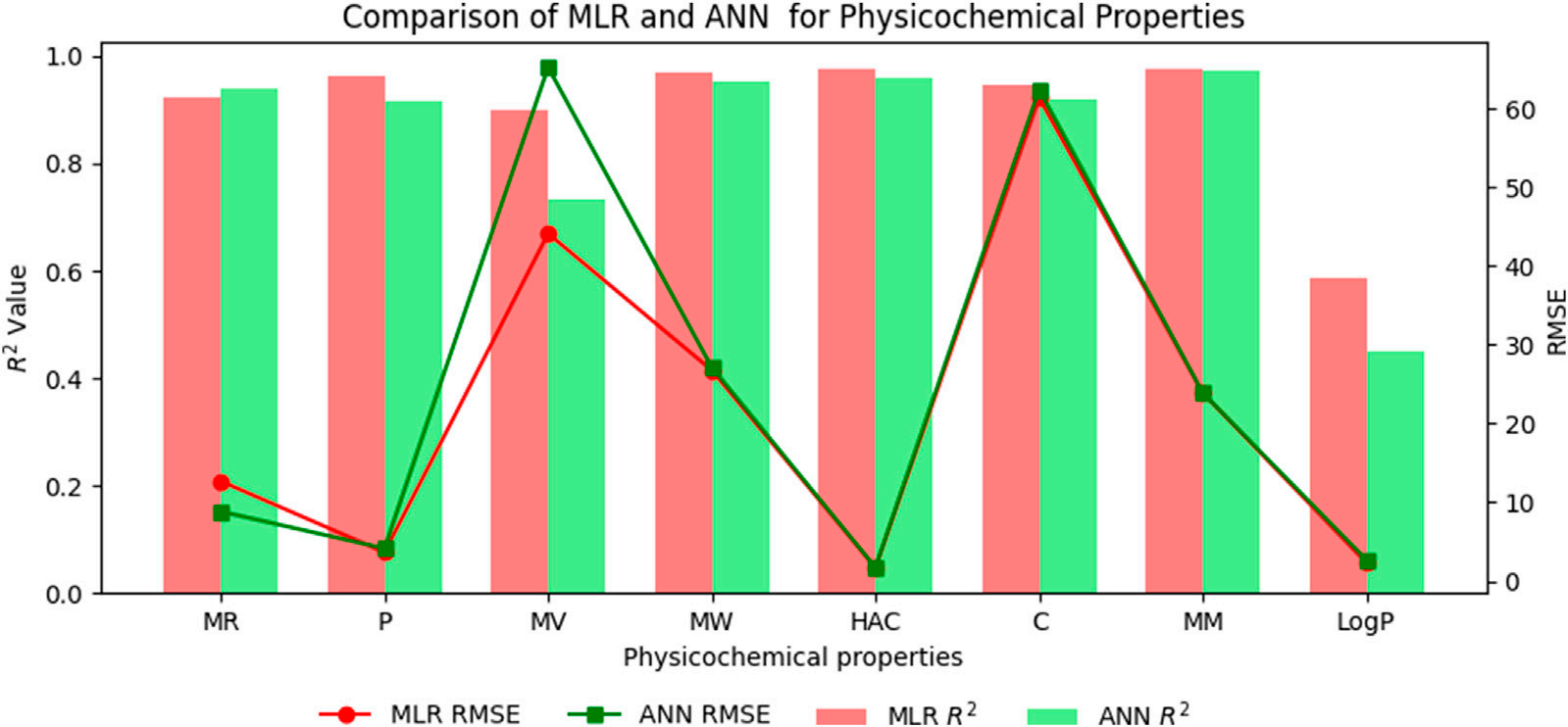
Comparison graph between MLR and ANN for physicochmeical properties.

**FIGURE 7:**
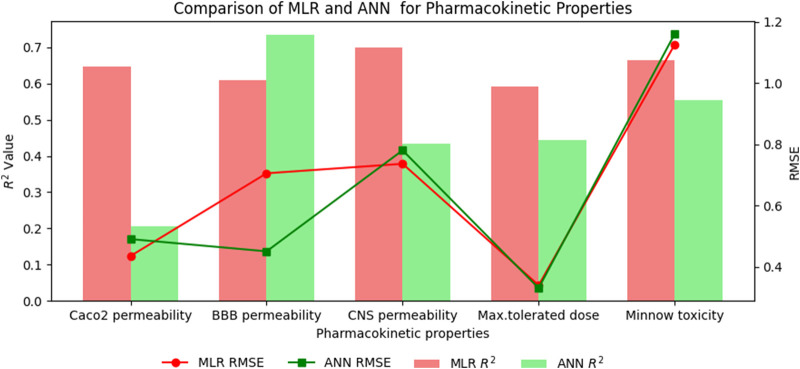
Comparison graph between MLR and ANN for pharmacokinetic properties.

An artificial neural network (ANN) is a computational system inspired by the human brain, consisting of interconnected artificial neurons. Recognized for its ability to uncover complex nonlinear relationships, the ANN is widely used for predictive purposes across various fields, often outperforming traditional models. In this section a comparison is carried out between multilinear regression and ANN model for physicochemical and pharmacokinetic properties of filovirus drugs using reverse sum Revan indices. From Tables 17, 18 it can be noted that the MLR provide high $R^{2}$ value and less standard error when compared to ANN model except for Molar Refractivity and BBB permeability. The comparison graph between MLR and ANN for physicochmeical and pharmacokinetic properties are shown in Figures 6, 7.

**TABLE 17:** Comparison of MLR and ANN modelling technique for physicochemical properties.

Physicochemical properties	MLR		ANN	
	R^2^	RMSE	R^2^	RMSE
MR	0.923	12.69428	0.940	8.84
P	0.963	3.64659	0.914	4.15
MV	0.898	44.14312	0.734	65.26
MW	0.968	26.76138	0.952	27.14
HAC	0.976	1.67835	0.959	1.74
C	0.945	61.39844	0.918	62.41
MM	0.974	23.88981	0.972	23.90
LogP	0.587	2.32452	0.448	2.70

**TABLE 18:** Comparison of MLR and ANN modelling technique for physicochemical properties.

Pharmacokinetic properties	MLR		ANN	
	R^2^	RMSE	R^2^	RMSE
Caco2 permeability	0.647	0.43488	0.207	0.49
BBB permeability	0.610	0.70498	0.735	0.45
CNS permeability	0.699	0.73604	0.435	0.78
Max.tolerated dose	0.591	0.33918	0.445	0.33
Minnow toxicity	0.665	1.12539	0.555	1.16

### 8.2 Results and discussion

The comparison of the performance of multilinear regressions (MLR) and artificial neural network (ANN) modelling technique for predicting physicochemical and pharmacokinetic properties of anti-filovirus drugs is highlighted through Tables 17, 18. The analysis is based on the $R^{2}$ values (which indicate how well the model fits the data) and the RMSE values (which measure the standard deviation of prediction errors). Here, we summarize which modelling technique showed superior performance for each property.

(i). Molar Refractivity (MR): The ANN outperformed MLR with a higher $R^{2}$ (0.940) and a lower RMSE (8.84) compared to the MLR ( $R^{2}$ = 0.923, RMSE = 12.69). This indicates that ANN captured the nonlinear relationships more effectively.
(ii). Polarizability (P): MLR provided a better fit with a higher $R^{2}$ (0.963) and a lower RMSE (3.65) compared to the ANN model ( $R^{2}$ = 0.914, RMSE = 4.15).
(iii). Molar Volume (MV): MLR performed better, showing a higher $R^{2}$ (0.898) and significantly lower RMSE (44.14) than ANN ( $R^{2}$ = 0.734, RMSE = 65.26).
(iv). Molecular Weight (MW): The $R^{2}$ values were similar between the both modelling technique (MLR: 0.968, ANN: 0.952), but MLR had a slightly better RMSE (26.76 vs. 27.14).
(v). Heavy Atom Count (HAC): MLR slightly outperformed ANN with a higher $R^{2}$ (0.976 vs. 0.959) and lower RMSE (1.68 vs. 1.74).
(vi). Complexity (C): MLR demonstrated better performance with $R^{2}$ of 0.945 and RMSE of 61.40 compared to ANN ( $R^{2}$ = 0.918, RMSE = 62.41).
(vii). Monoisotopic Mass (MM): Both models performed comparably, with MLR showing a marginally better RMSE (23.89) than ANN (23.90).
(viii). LogP: MLR outperformed ANN with a higher $R^{2}$ (0.587) and a lower RMSE (2.32), compared to ANN ( $R^{2}$ = 0.448, RMSE = 2.70).
(ix). Caco2 Permeability: MLR had a higher $R^{2}$ (0.647) than ANN (0.207) and a similar RMSE (0.43 for MLR vs. 0.49 for ANN), indicating better performance by MLR.
(x). BBB Permeability: ANN showed superior performance with a higher $R^{2}$ (0.735) and lower RMSE (0.45) compared to MLR ( $R^{2}$ = 0.610, RMSE = 0.70), suggesting better predictive capability for this property.
(xi). CNS Permeability: MLR outperformed ANN with a higher $R^{2}$ (0.699) and lower RMSE (0.73) than ANN ( $R^{2}$ = 0.435, RMSE = 0.78).
(xii). Maximum Tolerated Dose: The ANN model had a lower RMSE (0.33) but a lower $R^{2}$ (0.445) compared to MLR ( $R^{2}$ = 0.591, RMSE = 0.34).
  (xiii). Minnow Toxicity: MLR had a higher $R^{2}$ (0.665) and lower RMSE (1.13) compared to ANN ( $R^{2}$ = 0.555, RMSE = 1.16).

The multilinear regressions generally showed stronger or comparable performance for both physicochemical and pharmacokinetic properties, with lower prediction errors and higher $R^{2}$ values than ANN modelling technique. Thus, providing a benchmark for the performance of the newly introduced reverse sum Revan indices.

## 9 Conclusion and future work

This article introduced a new degree based topological indices, known as reverse sum Revan indices, and computed them for the isomorphic molecular graphs of anti-filovirus drugs considered in this research. A Quantitative Structure-Property Relationship (QSPR) analysis using multilinear regression was performed to investigate the relationship between these indices and the physicochemical and pharmacokinetic properties of the drugs. When comparing the proposed reverse sum Revan indices to the existing reverse and Revan degree-based indices, we found that our new indices exhibit strong correlations with nearly all properties of anti-filovirus drugs. Additionally, the errors associated with our proposed indices are significantly reduced. This highlights the effectiveness of reverse sum Revan topological indices in predicting the physicochemical and pharmacokinetic properties of anti-filovirus drugs compared to traditional degree based indices. Additionally, the study suggests that theoretical analyses can help pharmaceutical industries to predict various properties of anti-filovirus drugs without relying on experimental testing. A comparative analysis of the Multilinear Regression (MLR) and Artificial Neural Network (ANN) models revealed that the multilinear regressions are more effective in predicting the properties of anti-filovirus drugs.

Despite significant progress occurring in the field of drug design, the primary methodology still relies on the utilization of topological descriptors. As a future work, these indices can provide valuable insights into the Quantitative Structure-Activity Relationship (QSAR) and Quantitative Structure-Toxicity Relationship (QSTR) analyses of different anti-filovirus drugs, anti-cancer drugs and other viral pathogens. Moreover, it would be interesting to explore the properties of various dendrimers, nanostructures, and chemical networks through reverse sum Revan indices. These indices can also be employed in Metal-Organic Frameworks (MOFs) to analyze and predict the properties of MOFs, aiding in their design, optimization, and application.

Beyond anti-filovirus drugs, these indices hold promise for a wide range of therapeutic areas. For instance, they could aid in analysis of chemical structures of cancer drugs and other viral pathogens, assisting in the rapid evaluation of candidate compounds based on their structural properties and pharmacokinetics, which is particularly valuable for emerging infectious diseases. Overall, the broader implications of the reverse sum Revan indices extend well beyond anti-filovirus drugs, providing valuable insights across multiple domains in drug discovery and material science. By incorporating these indices into future research, scientists can advance the development of more effective therapeutic strategies for a variety of diseases.

## Data Availability

The original contributions presented in the study are included in the article/Supplementary Material, further inquiries can be directed to the corresponding author.
