# Supplementary figures and images for: New reverse sum Revan indices for physicochemical and pharmacokinetic properties of anti-filovirus drugs
Source: Front Chem. 2024 Dec 19;12:1486933. doi: 10.3389/fchem.2024.1486933 (PMC11693449; doi:10.3389/fchem.2024.1486933)

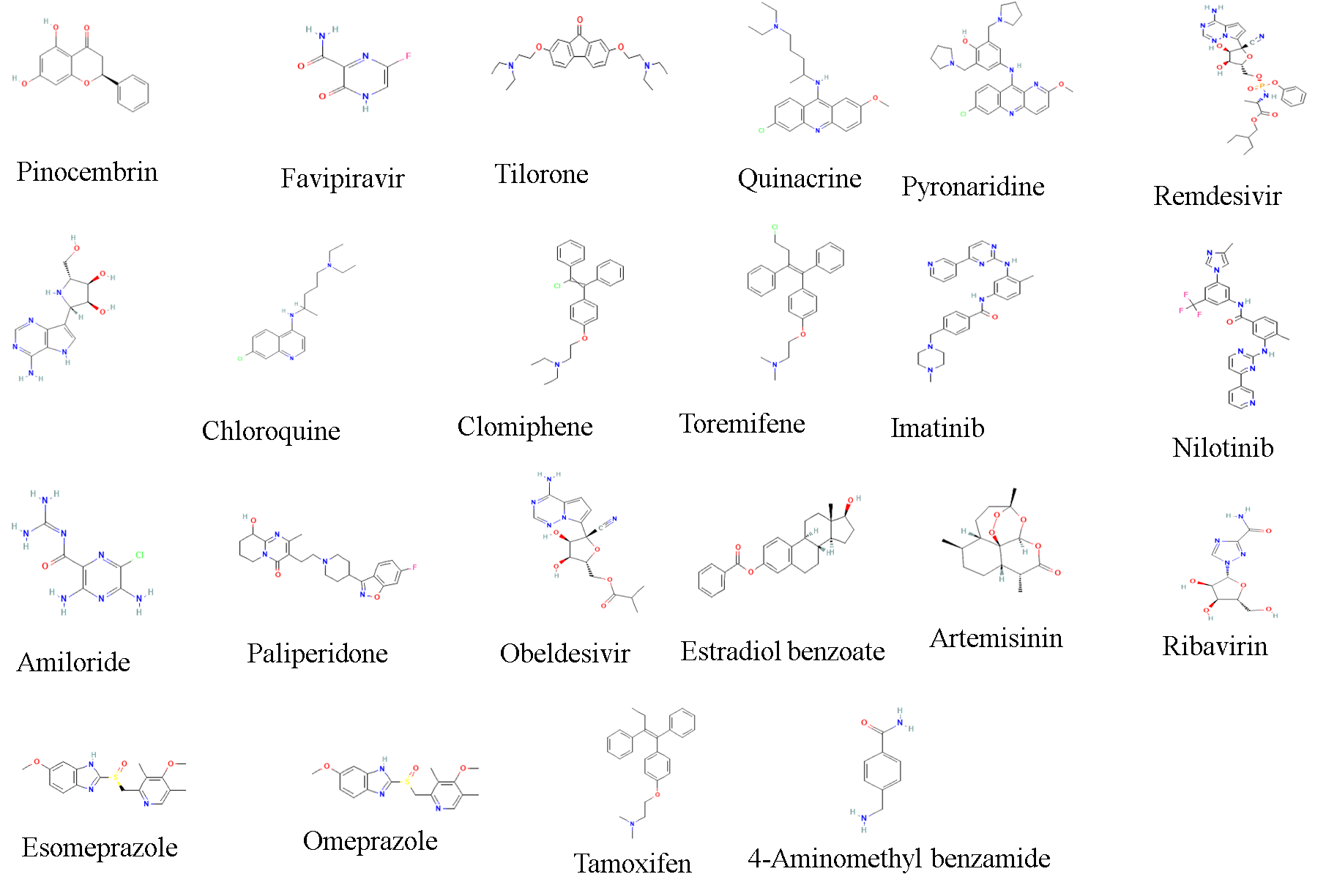

Supplement: Supplementary file 1 [file Image2.tif]

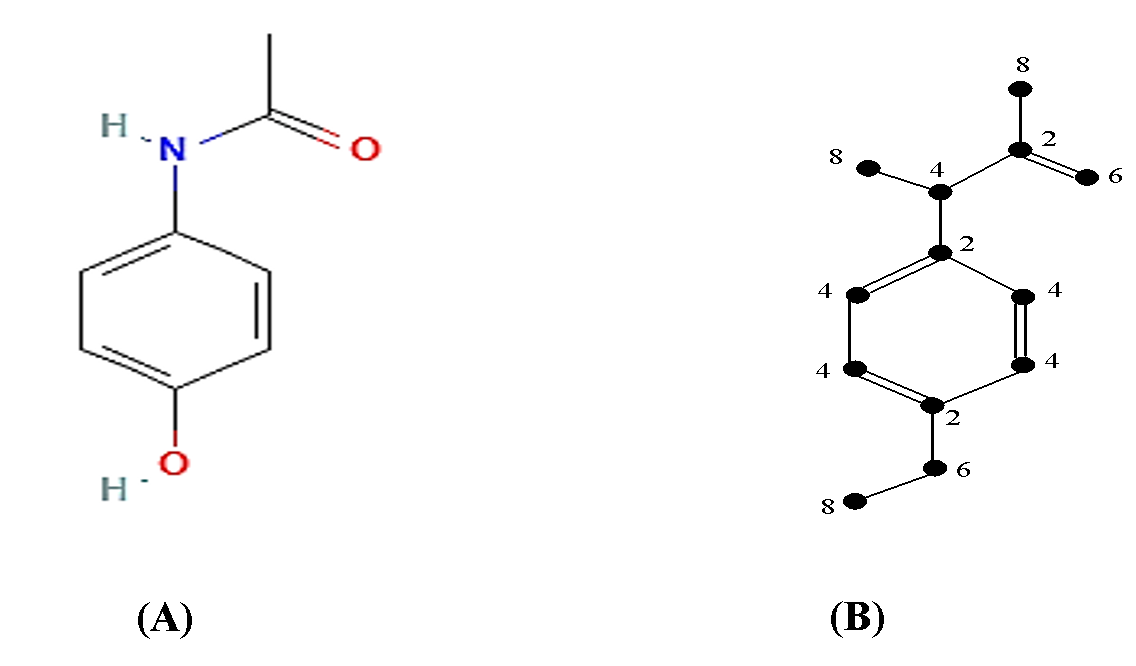

Supplement: Supplementary file 2 [file Image1.tif]
